# Involvement of the reticulospinal tract in the pathogenesis of spasticity after stroke and spinal cord injury

**DOI:** 10.3389/fnins.2026.1753609

**Published:** 2026-03-27

**Authors:** Sachiko Lee-Hotta

**Affiliations:** Division of Creative Physical Therapy, Department of Integrated Health Sciences, Graduate School of Medicine, Nagoya University, Nagoya, Japan

**Keywords:** neuronal plasticity, reticular formation, reticulospinal tract, spasticity, spinal cord injury, stroke

## Abstract

Spasticity occurs due to central nervous system (CNS) injuries such as stroke, spinal cord injury, cerebral palsy, or multiple sclerosis. Following CNS injury, spasticity does not appear immediately but emerges several days to weeks later and is believed to be the result of maladaptive changes due to neuroplastic alterations. Previously, research on the pathophysiology of spasticity has primarily focused on the spinal cord, leading to the elucidation of its mechanisms. Although the involvement of upper motor neurons has been suggested, definitive studies on the underlying pathophysiology have not been reported. In recent years, brainstem reticular formation has been identified as a region undergoing active plastic changes following a CNS injury. Although evidence remains limited and controversial, increasing numbers of studies suggest that plastic changes in brainstem reticular formation may be involved in the onset of spasticity. This review outlines the pathophysiological similarities and differences in spasticity between stroke and spinal cord injury, while summarizing recent studies on plastic changes in brainstem reticular formation and the onset of spasticity, including studies of humans and primates, as well as rodents. It organizes and examines the points of contention regarding the differences in outcomes between these two groups.

## 1 Introduction

Spasticity is characterized by an exaggeration of stretch reflex secondary to hyperexcitability of spinal reflexes and causes involuntary muscle contractions induced by joint movements and/or muscle stretching (Lance, 1980). Spasticity is classified as a symptom of upper motor neuron syndrome originating in the brain and brainstem, with projections to lower motor neurons within the brainstem and spinal cord (Adams and Hicks, 2005). Spasticity does not appear immediately after central nervous system (CNS) injury, but rather emerges several days or weeks later (Wissel et al., 2013). Spasticity is experienced following stroke and spinal cord injury (SCI) in approximately 20%–40% and 65%–78% of individuals, respectively (Maynard et al., 1990; Skold et al., 1999; Sommerfeld et al., 2004; Adams and Hicks, 2005; Sommerfeld et al., 2012). Spasticity is thought to be caused by plastic neural changes following injury. Previously, research on the pathophysiology of spasticity has primarily focused on the spinal cord, leading to the elucidation of its mechanisms. Although the involvement of upper motor neurons has been suggested, definitive studies on the underlying pathophysiology have not been reported. This review summarizes the pathways of the reticulospinal tract as a foundation for discussing its plastic changes. It then outlines the differences between stroke and SCI in terms of the effects of plastic changes occurring in the reticulospinal tract on motor function and spasticity. Furthermore, focusing specifically on stroke and SCI, the review summarizes the respective overviews of neural circuit damage and differences and similarities in spasticity pathophysiology, proposing hypotheses due to differences in the involvement of the reticulospinal tract in spasticity.

## 2 Reticulospinal tract

**Figure 1:**
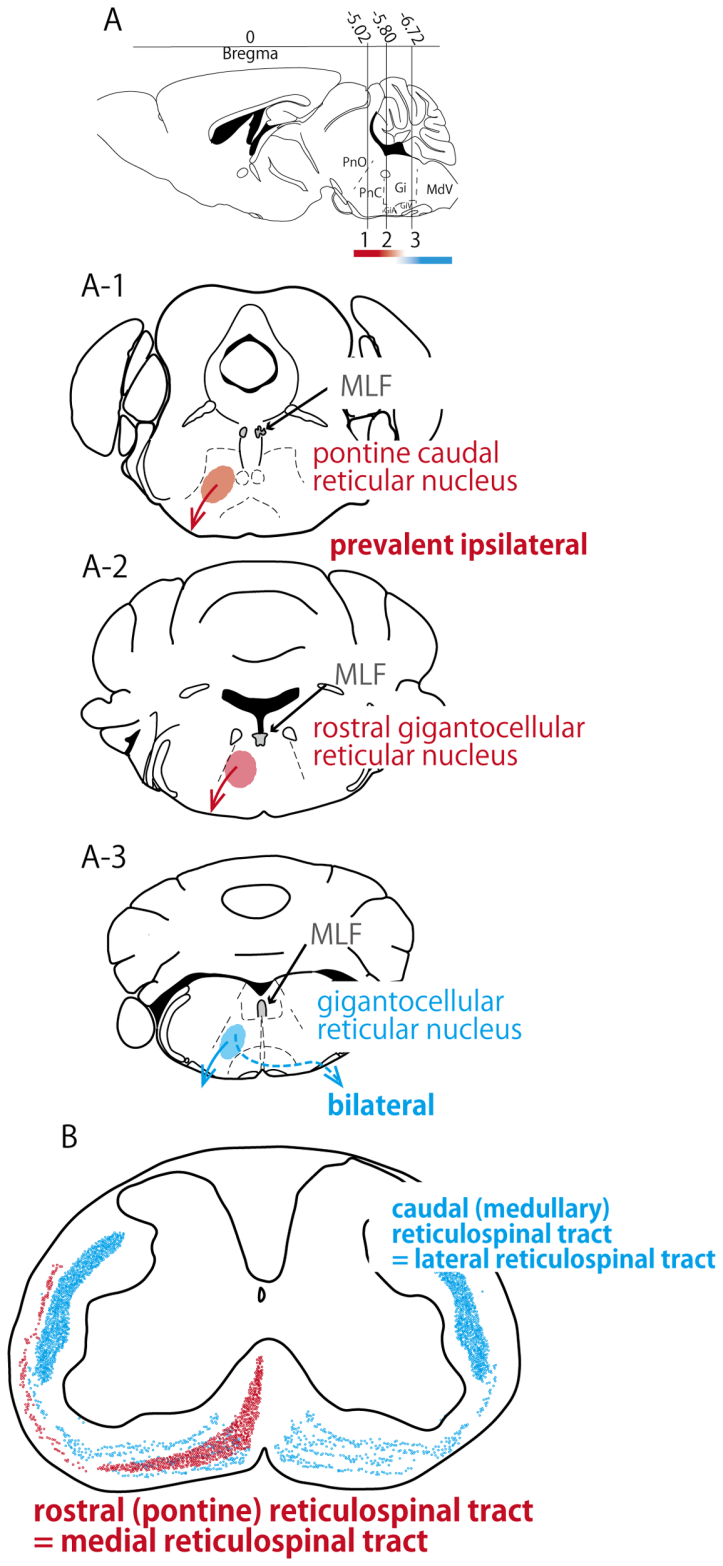
Localization of medial, lateral reticulospinal tracts and medial longitudinal fasciculus in mice at the cervical level. **(A)** Sagittal plane of mouse brain. Lines 1, 2, and 3 indicate the levels of the cross sections of **(A-1–A-3)**, respectively. **(B)** Cross section of cervical spinal cord (Watson and Harrison, 2012). PnO, Pontine reticular nucleus, oral part; PnC, Pontine reticular nucleus, caudal part; Gi, Gigantocellular reticular nucleus; GiA, Gigantocellular reticular nucleus, alpha part; GiV, Gigantocellular reticular nucleus, ventral part; MdV, medullaly reticular nucleus, ventral part.

The reticulospinal tract is one of the most important extrapyramidal tracts and constitutes the major descending motor pathway monosynaptically connecting to motor neurons (Esposito et al., 2014). The regions that form the main reticulospinal tracts are the pontine reticular formation and medullary reticular formation (Figure 1). In general, the nuclei of the pontine reticular formation project ipsilaterally to the spinal cord via the ventromedial portion of their white matter and are excitatory neurons that facilitate their projection sites. Conversely, the nuclei of the medullary reticular formation project bilaterally to the spinal cord via its lateral portion and form either excitatory or inhibitory connections with motor neurons and propriospinal neurons (Rhines and Magoun, 1946; Peterson et al., 1978; Peterson et al., 1979; Mitchell et al., 2016). Moreover, many of these tracts exhibit long-distance projections, giving off branches across a broad expanse of the spinal cord from the cervical to the lumbar regions (Peterson et al., 1975). This pathway is largely identical in humans and rodents, and is known as the medial reticulospinal tract in primates and the rostral reticulospinal tract in rodents. The nuclei of the medullary reticular formation descend laterally to the white matter and project to the left and right spinal cords. Similar to the pontine reticulospinal tract, the medullary reticulospinal tract forms comparable pathways in humans and rodents, termed the lateral reticulospinal tract in humans and caudal reticulospinal tract in rodents (Figure 1) (Watson and Harrison, 2012; Haines et al., 2019). However, a detailed study examining the spinal pathways originating from the pontomedullary gigantocellular (Gi) reticular nucleus corresponding to the medial reticulospinal tract revealed bilateral ventral and lateral funiculi with an ipsilateral predominance in the spinal cord (Liang et al., 2016). Therefore, considerable variation exists in each brainstem reticular nucleus and spinal cord. In addition, the reticulospinal tract projecting from the reticular formation comprises diverse nuclei within the brainstem, such as the pons and medulla, to spinal cord, and it is known that in rodents, the primary origin in quantitative terms is the Gi reticular nucleus located within the medulla (Liang et al., 2011; Wang et al., 2022).

The reticular formation plays a central role in regulating posture, muscle tone, and spinal reflexes, controlling proximal and axial muscles, and gross movements such as locomotion and reaching (Magoun, 1944; Magoun and Rhines, 1946; Rhines and Magoun, 1946; Siegel, 1979; Mori, 1987; Baker, 2011). In humans and primates, the corticospinal tract is responsible for controlling fine motor movements because it connects directly to spinal motor neurons, whilst the reticulospinal tract plays a supplementary role. In particular, in rodents, the reticulospinal tract is considered one of the primary descending motor pathways involved in motor function, alongside the corticospinal tract. It controls not only posture and muscle tone, but also forelimb skilled movement (Esposito et al., 2014), and locomotor function such as stopping (Bouvier et al., 2015), speeding (Capelli et al., 2017; Zhang et al., 2024), and turning (Cregg et al., 2020; Usseglio et al., 2020) during gait and running. In humans and primates, the nuclei of the brainstem reticular formation primarily regulate muscle tone and postural control rather than direct motor generation. In contrast, the rodent nuclei of the brainstem reticular formation utilize various neurotransmitters, including excitatory glutamatergic neurons, inhibitory glycinergic and/or GABAergic neurons, and monoaminergic neurons, and contribute to direct motor control (Ruder and Arber, 2019). These species-dependent differences suggest that plastic changes in the reticulospinal tract may have distinct functional consequences on spasticity. Furthermore, the motor neurons to which the reticulospinal tract projects and connects may exhibit a bias towards the flexor/extensor muscles. Results from studies using monkeys showed that the pontomedullary reticulospinal tract tends to facilitate flexors and suppress extensors ipsilaterally, and facilitate extensors and suppress flexors contralaterally (Davidson and Buford, 2006; Davidson et al., 2007). Flexor/extensor bias may be observed in muscles that are prone to developing spasticity.

The reticular formation has recently been reported to cause dynamic plastic neural changes and contribute to the recovery of motor function after stroke and SCI (Akalu et al., 2023). Moreover, recent thinking is that plastic neural changes in reticular formation after injury might be related to the onset of spasticity; however, there may be differences depending on the injury, such as stroke and SCI. Next, we describe the role of plastic neural changes in the reticulospinal pathway after stroke and SCI in humans and primates and in rodents.

## 3 Plastic neural changes of the reticulospinal tract after stroke and/or corticospinal lesions in humans and primates

In the reticulospinal tract and subcortical pathways, plastic neural changes related to motor recovery have been reported to occur following stroke in many species, including humans, monkeys, and rodents (Zaaimi et al., 2012; Bachmann et al., 2014; Akalu et al., 2023; Isumi et al., 2024). Zaaimi et al. (2012) investigated which descending motor pathway connects to motor neurons after recovery from corticospinal lesions in macaque monkeys using intracellular recordings from motor neurons innervating the hand and forearm muscles. Descending motor pathways were activated via electrode stimulation of the unlesioned (ipsilateral) pyramidal tract for the corticospinal tract and the ipsilesional medial longitudinal fasciculus (MLF) for the medial reticulospinal tract. After recovery from the lesion, input from the ipsilateral pyramidal tract to intrinsic hand muscles was rarely observed or absent. In contrast, monosynaptic inputs from MLF stimulated by an electrode close to the middle pontine level were elicited in motor neurons projected to intrinsic hand muscles in lesioned animals, and the total input of MLF to intrinsic hand motor neurons increased 2.5-fold compared with control animals. In marked contrast to the substantial changes observed in the forearm flexor and intrinsic hand motor neurons, no consistent changes were observed in the forearm extensor motor neurons. Normally, the medial and lateral reticulospinal tracts (pontomedullary reticulospinal tract) in monkeys facilitate the ipsilateral flexors and contralateral extensors (Davidson and Buford, 2006). In these studies, the projections of the reticulospinal tract underwent plastic changes after the corticospinal tract injury, resulting in excessive connections to the spinal motor neurons projecting to the flexors of the forearm and the intrinsic hand muscles. This finding is particularly interesting for individuals exhibiting spasticity. Spasticity occurs more frequently in the flexor muscle groups of the upper extremities (Marciniak, 2011; Hefter et al., 2012). Xu et al. (2017) reported two critical aspects of hand function: strength and independent control of fingers for functional recovery after stroke. Most gains in strength and individuation occur within the first 3 months after stroke, with recovery in both domains closely correlated up to a strength level below 60% of the estimated premorbid strength. Beyond this, any further improvement in strength was not accompanied by further improvement in individuation. The authors considered the reticulospinal tract a strong candidate as the neuronal circuit that contributes to the recovery of muscle strength and initial dexterity. They pointed out that neuronal circuits distinct from those involved in the recovery of muscle strength and dexterity are required for subsequent improvement in dexterity (second-stage dexterity). Although these plastic changes support recovery, they may also predispose to abnormal muscle activation characteristic of spasticity.

The reticulospinal tract contributes to motor recovery and/or recovery of muscle strength after stroke and corticospinal injury (Akalu et al., 2023); however, one study reported that plastic changes occurring in the medial reticulospinal tract showed a negative correlation with the motor performance index (Karbasforoushan et al., 2019). They clarified that decreased integrity of the ipsilesional corticospinal tract and increased integrity of contralesional medial reticulospinal tract correlate with the severity of motor impairment in individuals with stroke by using high-resolution diffusion tensor imaging (DTI) based on magnetic resonance imaging (MRI) of both the brainstem and cervical spinal cord (Karbasforoushan et al., 2019). Furthermore, in the sub-acute phase in individuals with stroke, no evidence was reported of enhanced connectivity in the contralesional reticulospinal tract contributing to any components of impairment, such as muscle strength and synergies (Fugl-Meyer upper-limb score) (Hammerbeck et al., 2021). The authors investigated the contribution of the reticulospinal tract to motor recovery in individuals with stroke using transcranial magnetic stimulation of the contralesional motor cortex, M1. Although the contralesional motor cortex stimulation was used as a proxy for reticulospinal tract integrity, such as the ipsilateral cortico–reticulo–propriospinal pathway (Bradnam et al., 2013), the study did not employ stimulation methods that directly and specifically activate the reticulospinal tract. In general, detection of plastic changes within the reticulospinal tract requires direct activation of reticulospinal neurons, which is typically achieved by electrical stimulation of the medial longitudinal fasciculus (MLF). The MLF is known to contain a substantial proportion of medial reticulospinal fibers originating from the pontine reticular formation, rather than transcranial magnetic stimulation of the contralesional motor cortex (Watson et al., 2012; Haines et al., 2019).

**Figure 2:**
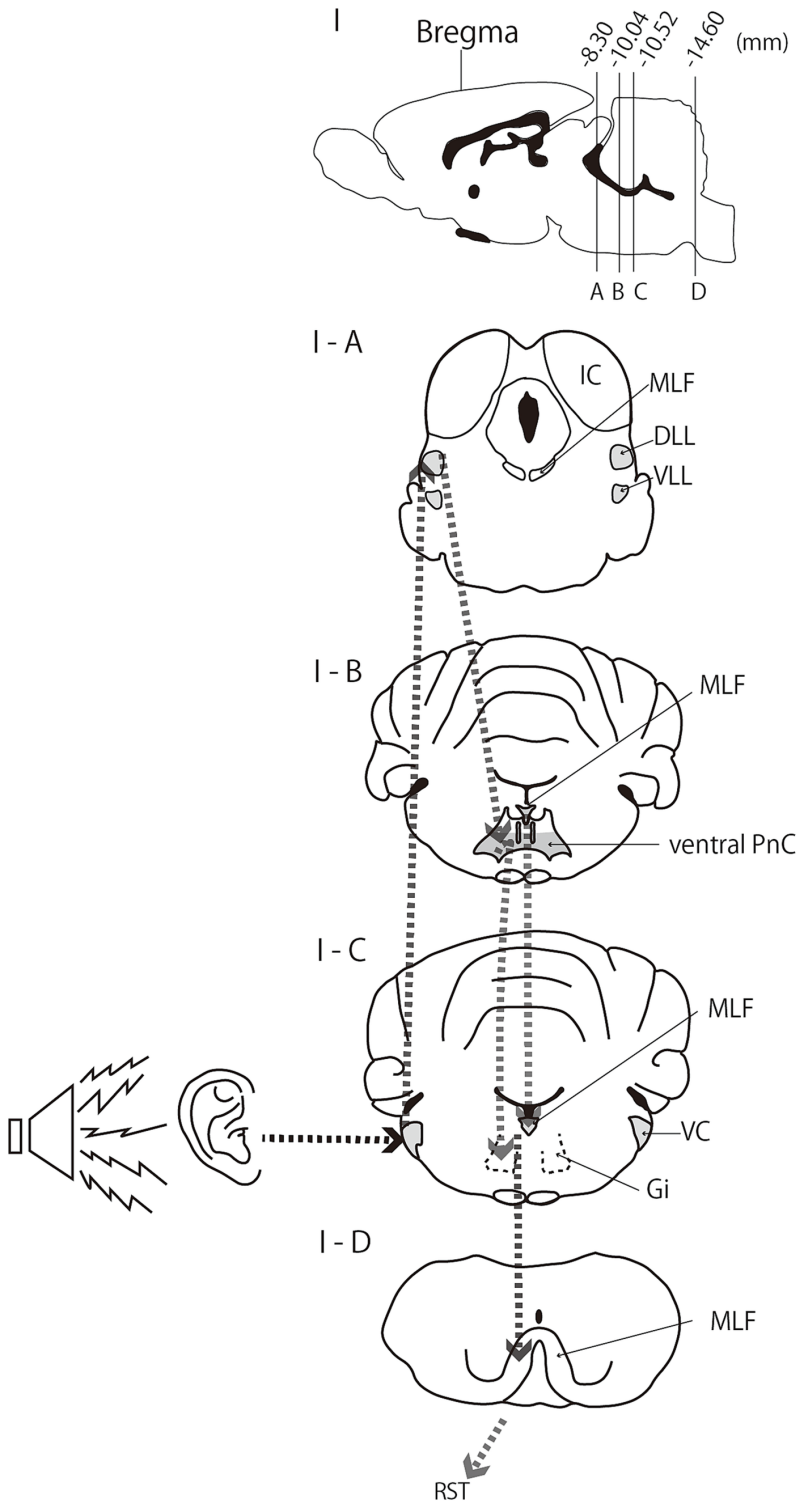
Acoustic startle reflex circuit in rats. **(I)** Sagittal section of the rat brain. **(I-A–D)** are coronal sections at positions 8.30, 10.04, 10.52, and 14.60 mm caudal to the bregma. IC, Inferior colliculus; MLF, medial longitudinal fasciculus; DLL, dorsal lateral lemniscus; VLL, ventral lateral lemniscus; PnC, ventral pontine reticular nucleus, caudal part; VC, ventral cochlear nucleus; Gi, gigantocellular reticular nucleus; RST, reticulospinal tract. The auditory nerve projects directly to the VC. The dotted arrows show input direction and the neural circuit from the auditory nerve stimulated by a loud sound. Modified from Davis et al. (1982), *Journal of Neuroscience*, 1982. Copyright 1982 Society for Neuroscience.

StartReact is widely used as an indirect probe of reticulospinal tract excitability in humans. The acoustic startle reflex, also known as the StartReact effect, is used as a noninvasive methodological tool to activate the reticulospinal tract in humans, given its established association with the medial reticulospinal tract. The acoustic startle reflex is an unconscious reaction in which the body automatically responds to sudden sounds. The StartReact response is quantified as the time between the startle-inducing cue and the onset of the response, as measured by the electromyogram burst in the responding muscle (Brown et al., 1991). The acoustic startle circuit in rodents has been shown to include the dorsal and ventral nuclei of the lateral lemniscus (DLL and VLL, respectively), ventral pontine reticular nucleus, caudal part (PnC), ventral cochlear nucleus (VC), and MLF, whereas the pontine reticular nucleus, oral part (PnO), Gi, and dorsal PnC were excluded (Davis et al., 1982) (Figure 2). Using StartReact, Li et al. (2014) investigated spasticity in chronic stroke patients and reported that 58.3% of the spasticity group showed prolonged acoustic startle reflex duration. However, no significant correlation was found between the severity of spasticity and the characteristics of startle responses within this group. In this study, activity of the medial reticulospinal tract, including the MLF activated by the StartReact response, was associated with the presence of spasticity.

## 4 Plastic neural change of the reticulospinal tract after stroke in rodents

Studies on plastic changes in the reticulospinal tract after stroke in rodents are limited, and some studies have yielded conflicting results regarding whether the reticulospinal tract is involved in the recovery of motor function. However, whether similar mechanisms operate in rodents remains controversial.

Widespread spontaneous plastic changes have been reported in different brainstem nuclei by unilateral retrograde tracing from the affected cervical spinal cord, which was devoid of corticospinal input, 4 weeks after stroke in mice (Bachmann et al., 2014). Several parts of the medullary reticular formation constituting lateral reticulospinal tract, as well as the spinally projecting raphe nuclei, increased their projections to the cortically denervated affected cervical cord by 1.2- to 1.6-fold. Furthermore, plastic changes in the cortico-brainstem pathway using an internal capsule hemorrhage stroke model in rats reported abundant sprouting of motor cortex axons in the red nucleus, but not in the medullary reticular formation, during the early recovery phase in rehabilitated rats. Moreover, the authors revealed that cortico-rubral tract blockade from the start of rehabilitation induced an obvious increase in axon sprouting in the medullary reticular formation, with substantial functional recovery (Ishida et al., 2019).

In contrast, 4 weeks after photothrombotic stroke in the rostral and caudal regions of the mouse forelimb, no spontaneous plastic changes in the amount of axons projecting to spinal neurons were observed when retrograde tracers were injected into the affected cervical spinal cord to visualize brainstem neurons projecting to spinal neurons, except within the ipsilesional Gi reticular nucleus constituting lateral reticulospinal tract (Okabe et al., 2018). The authors reported that rehabilitation-induced functional recovery did not occur in the brainstem regions, including the rubrospinal and both medial and lateral reticulospinal tracts, as these circuits seemed unable to compensate for the function of the corticospinal tract (Okabe et al., 2018; Okabe and Miyamoto, 2018). Although differences in injury models may have contributed to the conflicting results, spontaneous plasticity is believed to occur within the brainstem regions. In general, for the analysis of projection volume via axonal sprouting in spontaneous plastic changes, it is necessary to visualize axon terminals in the anterograde direction. However, whether these neuroplastic changes contribute to the recovery of motor function remains unclear. Notably, Ishida et al. demonstrated evidence of functional improvement using a viral vector in a loss-of-function model, suggesting that brainstem plasticity may support recovery to a limited degree (Ishida et al., 2019).

## 5 Plastic neural changes in the reticulospinal tract after spinal cord injury

In the plastic changes that occur in the reticular formation have been reported to interact with the onset of spasticity rather than with the recovery of motor function in individuals with SCI. Sangari and Perez (2019) compared responses in the lower limb rectus femoris muscle using motor–evoked potentials (MEPs) elicited by transcranial magnetic stimulation (TMS) on the primary motor cortex, maximal voluntary contractions (MVCs), and the StartReact response between individuals with and without spasticity after incomplete SCI, and between healthy individuals. As a result, in SCI individuals with spasticity, compared with healthy subjects and SCI individuals without spasticity, MEPs and MVCs were smaller, and StartReact responses were larger. That is, individuals with SCI exhibiting spasticity, as measured by the Modified Ashworth Scale (MAS) clinical score of 2–4, showed a negative correlation between this score and both MVCs and MEPs, whilst exhibiting a positive correlation with the StartReact response medial reticulospinal tract. Furthermore, the authors confirmed the involvement of the corticospinal and reticulospinal tracts in the spontaneous contractions of the flexors and extensors of the upper extremities after cervical SCI. Regarding the involvement of the corticospinal tract, TMS-induced MEPs and MVCs in the flexor muscles and, biceps brachii showed no differences between healthy subjects and individuals with SCI. In contrast, in the triceps brachii, both MVCs and MEPs were significantly reduced in individuals with SCI compared with healthy subjects, confirming reduced connectivity of the corticospinal tract to extensor muscles. Regarding the medial reticulospinal tract, an enhancement of the StartReact response was observed in individuals with SCI for the flexor muscle, the biceps brachii. However, for the extensor muscle, the triceps brachii, no change was observed compared with healthy subjects. In other words, the development of a compensatory neural circuit via the medial reticulospinal tract was confirmed for flexor muscles in SCI (Sangari and Perez, 2020). In contrast to studies in humans focusing on spasticity, studies in rodents have mainly focused on motor recovery.

Studies using SCI models for hemisection and contusion in rodents have reported many instances where plastic changes in the lateral reticulospinal tract form compensatory circuits that contribute to motor recovery. This is particularly evident in projections from the brainstem reticular nuclei to the spinal cord, including the ipsilesional Gi (Filli et al., 2014), contralesional Gi (Zorner et al., 2014; Engmann et al., 2020), and ventral Gi (Asboth et al., 2018), where the number of neurons and axons projecting to the spinal cord increases. Furthermore, reports indicate that not only intact reticulospinal fibers but also damaged fibers form collateral branches, establishing compensatory neural circuits via spinal cord intrinsic neurons, thereby contributing to improved motor function (May et al., 2017). Despite the significant improvement in locomotion ability as part of motor functional recovery, impairment in skilled movements of the forelimbs persists. Regarding the spasticity symptoms mentioned in human SCI, Asboth et al. (2018) reported that when comparing a training group that underwent automatic stepping on the treadmill with gravity assist and/or robot-assisted walking training with a non-training group after SCI due to contusion in the thoracic spinal cord, simultaneous contraction of the primary agonist and antagonist muscles was observed in the non-training group, whereas reciprocal activity was observed in the training group. Although axonal projections from the reticular formation beyond the spinal cord lesion were observed in the non-training group, similar to intact rats, these reticulospinal tract axonal projections significantly increased beyond the lesion in the training group. Other studies have not evaluated spasticity in this context, and there is scarcely any mention of them. In other words, in SCI studies using rodents, the interaction between increased plastic changes in the lateral reticulospinal tract and spasticity is currently unclear or is thought to be weak. At present, as mentioned above, a contradiction persists in SCI research: whilst studies in humans indicate that plastic changes in the medial reticulospinal tract correlate with spasticity symptoms (Sangari and Perez, 2019, 2020), research in rodents shows that plastic changes in this tract improve motor function, yet little mention is made of any association with spasticity (Filli et al., 2014; Zorner et al., 2014; Asboth et al., 2018; Engmann et al., 2020). In both cases, plastic changes occur in the reticulospinal tract. However, a significant difference lies in the fact that the functions performed by the brainstem reticular nuclei differ between humans and primates on the one hand and rodents on the other. The brainstem reticular nuclei in humans and primates are known to play a secondary role in motor control, such as posture regulation and muscle tone control. Therefore, this role could also be interpreted as an exaggerated manifestation resulting from plastic changes occurring in brainstem reticular formation. Furthermore, regarding the relationship between plastic changes in the reticular nuclei of the brainstem of rodents and spasticity in SCI, currently, no reports have conducted on spasticity assessments. In rodents, the assessment of spasticity using Hoffmann’s reflex is commonly used. Indeed, in mice with cervical spinal cord hemisection SCI, a reduction in the reflex’s RDD, indicative of spasticity, has been observed at 4 weeks post-SCI (Hanasaki et al., 2025). However, this report did not observe changes in the reticulospinal tract. Therefore, whilst acknowledging the need for continued research, it appears that the reticular nuclei of the brainstem in rodents play a more central role in motor execution than in humans or primates. Consequently, species-dependent differences in SCI outcomes may be interpreted as reflecting variations in the function of the reticular nuclei of the brainstem. In addition to these functional differences, species-dependent outcomes may also be influenced by anatomical differences in the organization of the reticulospinal system. Quantitative anatomical analyses in rodents have demonstrated that the lateral reticulospinal tract, originating from the medullary reticular nuclei, constitutes the dominant descending projection within the reticulospinal system. In humans and primates, reports of plastic changes in the medial reticulospinal tract, which provides a predominantly excitatory input to the spinal cord, are relatively numerous, suggesting that this tract may exert a greater influence on spinal motor circuits (Rhines and Magoun, 1946). In contrast, in rodents, plastic changes in the lateral reticulospinal tract, which contains both excitatory and inhibitory projections, may have a stronger impact (Magoun and Rhines, 1946). Such differences in the relative contribution of the medial and lateral reticulospinal pathways may partially explain the divergent experimental findings on the relationship between reticulospinal plasticity, motor recovery, and spasticity between species.

## 6 The pathophysiological mechanisms in spinal cord spasticity following stroke and SCI

**Figure 3:**
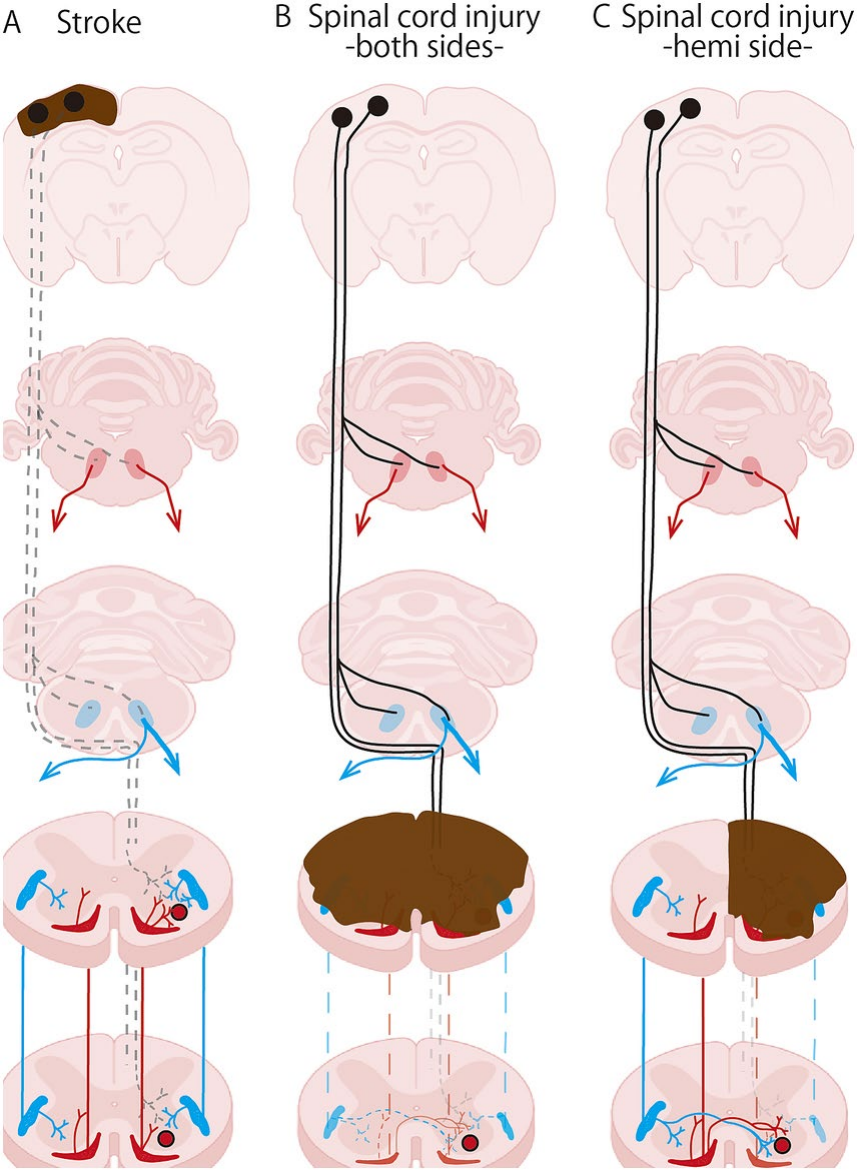
Schematic diagram of a damaged neural pathway due to stroke and spinal cord injury in rodents. **(A)** Stroke with damage to cortical neurons, **(B)** Nearly complete damage to both sides of the spinal cord, **(C)** Incomplete damage to hemi side of the spinal cord. Filled black circle: cortical neurons, black solid line: corticospinal tract, pale red circle: rostral reticular nucleus, pale blue circle: caudal reticular nucleus, red solid line: rostral reticulospinal tract, blue solid line: caudal reticulospinal tract, brown area: injury site, dotted lines: damaged pathways.

To better interpret these supraspinal plastic changes, understanding the overview of neuronal and neural pathways injuries in stroke and SCI will support understanding of the similarities and differences in the pathophysiological neural responses underlying spasticity. In stroke, ischemic stroke accounts for up to 65.3% of all stroke cases, followed by intracerebral hemorrhage, which constitutes 28.8% of all strokes (GBD 2021 Stroke Risk Factor Collaborators, 2024). In the most typical atherothrombosis, damage to cortical neurons occurs. In this case, the brainstem reticular formation remains unaffected. Moreover, the most common site of SCI in individuals aged 70 years and over is high cervical SCI (C1–C4) due to falls, accounting for 57% of cases. In contrast, thoracolumbar SCI due to accidents is more prevalent in younger individuals. Regarding severity, the most common American Spinal Injury Association (ASIA) grade at admission for individuals ≧70 years old was D, classified as an incomplete injury, indicating residual motor function below the level of neurological injury. Muscle strength of grade 3 or higher on the Muscle Manual Test (MMT) was observed in the majority of paralyzed areas, accounting for 56.8%. For individuals <70 years, the grade was classified as A, representing complete paralysis, with total loss of motor and sensory function in the S4/5 spinal cord region, affecting 42.5% of cases. Moreover, at the one-year follow-up, ASIA grade A injury for individuals <70 years, approximately 32.5% of individuals improved to below B grade (Gebeyehu et al., 2024). In cases of incomplete SCI, some neural pathways in the remaining intact region persist (Figure 3). In the reticulospinal tract, although it remains intact following a stroke, it sustains axonal damage in SCI. This difference is likely to influence the plastic changes occurring in the neurons of the reticular formation of the brainstem.

**Figure 4:**
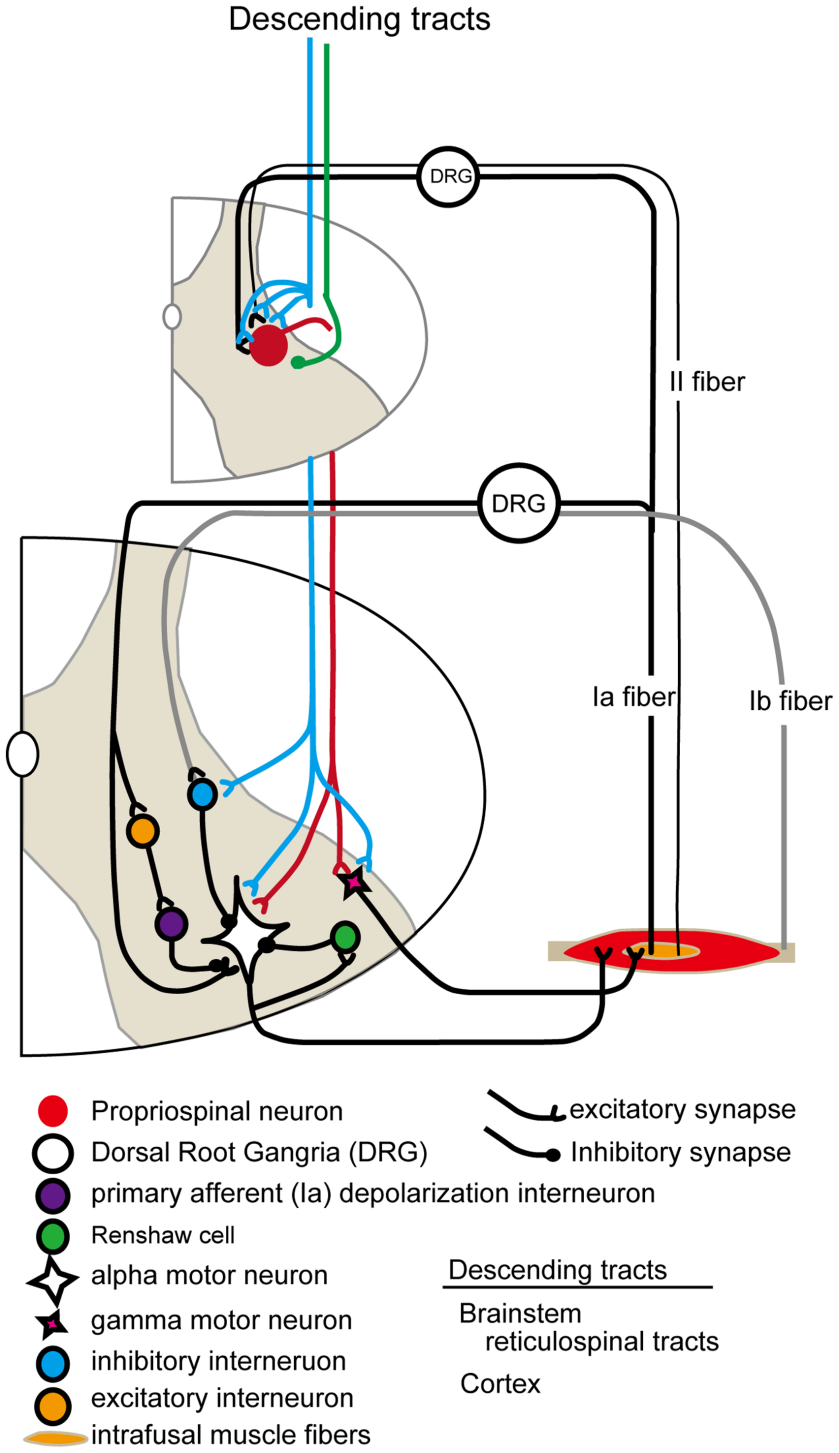
Schematic diagram of spinal neural circuits related to spasticity.

Mechanisms of spasticity in spinal cord levels after stroke and SCI show similarities and differences. An overview of spinal neural circuits associated with spasticity pathologies is shown in Figure 4. Previous studies have reported that motor neuron hyperactivity during the onset of spasticity is caused by the spontaneous generation of persistent inward currents (Gorassini et al., 2004; Murray et al., 2010). This is a pathological mechanism that arises from SCI that induces clonus, an abnormality caused by monoamine deficiency due to damage to the reticulospinal tract, and is rarely observed in stroke. In stroke and SCI mice, previous studies have reported that reduced expression of potassium chloride cotransporter 2 (Kcc2) on motor neuron membranes leads to impaired function of inhibitory receptors (gamma-aminobutyric acid A and glycine receptors), resulting in increased excitability of motor neurons. This Kcc2 expression recovers within 2 weeks post-onset in stroke, but remains unresolved even 6 months post-onset in SCI (Boulenguez et al., 2010; Toda et al., 2014; Lee-Hotta et al., 2019). Furthermore, several studies have demonstrated in individuals with stroke and SCI that the mechanism of spasticity involves signals from type Ia and type II afferent sensory nerves via the propriospinal neurons and spinal interneurons, which significantly promote excitatory input to motor neurons (Marque et al., 2001; Remy-Neris et al., 2003; Nardone and Schieppati, 2005). Reticulospinal fibers passing through the MLF connect to propriospinal neurons, comprising 73.7% vGluT2-positive excitatory fibers and 19.7% vGAT-positive inhibitory fibers. Propriospinal neurons also receive connections from corticospinal fibers. Although the number of connecting cells is greater for corticospinal fibers compared with reticulospinal fibers, the axon terminal density per 100 μm^2^ of cell surface was higher for reticulospinal fibers; approximately 100-fold higher for excitatory fibers at the cell body, and approximately 15 times higher for dendrites. For inhibitory fibers, the density is reported to be approximately 50 times higher for cell bodies and 4 times higher for dendrites, indicating a higher density than that of corticospinal fibers (Mitchell et al., 2016). Moreover, a spasticity mechanism involves a reduction in inhibitory spinal functions, such as pre- and post-synaptic inhibition in both stroke and SCI (Taylor et al., 1984; Aymard et al., 2000; Kapitza et al., 2012). The rate-dependent depression (RDD, known as post-activation depression) is a spasticity symptom observed in all diseases with the onset of spasticity. The Hoffman reflex serves as a method capable of sensitively confirming changes in the efficacy of Ia afferent synapses (Enriquez-Denton et al., 2002). The RDD is an important factor in the evaluation of stretch reflex excitability. These spinal mechanisms are strongly modulated by descending reticulospinal inputs.

## 7 Plastic changes in the medullary reticular formation ventral part in spasticity

The spasticity pattern of muscle involvement at onset tends to be similar across different underlying CNS injuries, such as stroke or SCI, with spasticity most commonly affecting the upper limb flexors, especially the fingers (Hefter et al., 2012) and the lower limb extensors (McDowell, 1981). Therefore, functional localization appears to be related to the pathophysiology of spasticity. The brainstem nucleus has a functional localization; that is, the spinal trigeminal nuclei, parvicellular reticular nuclei, medullary reticular nucleus ventral part (MdV) project to motor neurons innervating the flexors, and the vestibular nuclei project to motor neurons innervating the upper limb extensors in mice (Esposito et al., 2014).

**Figure 5:**
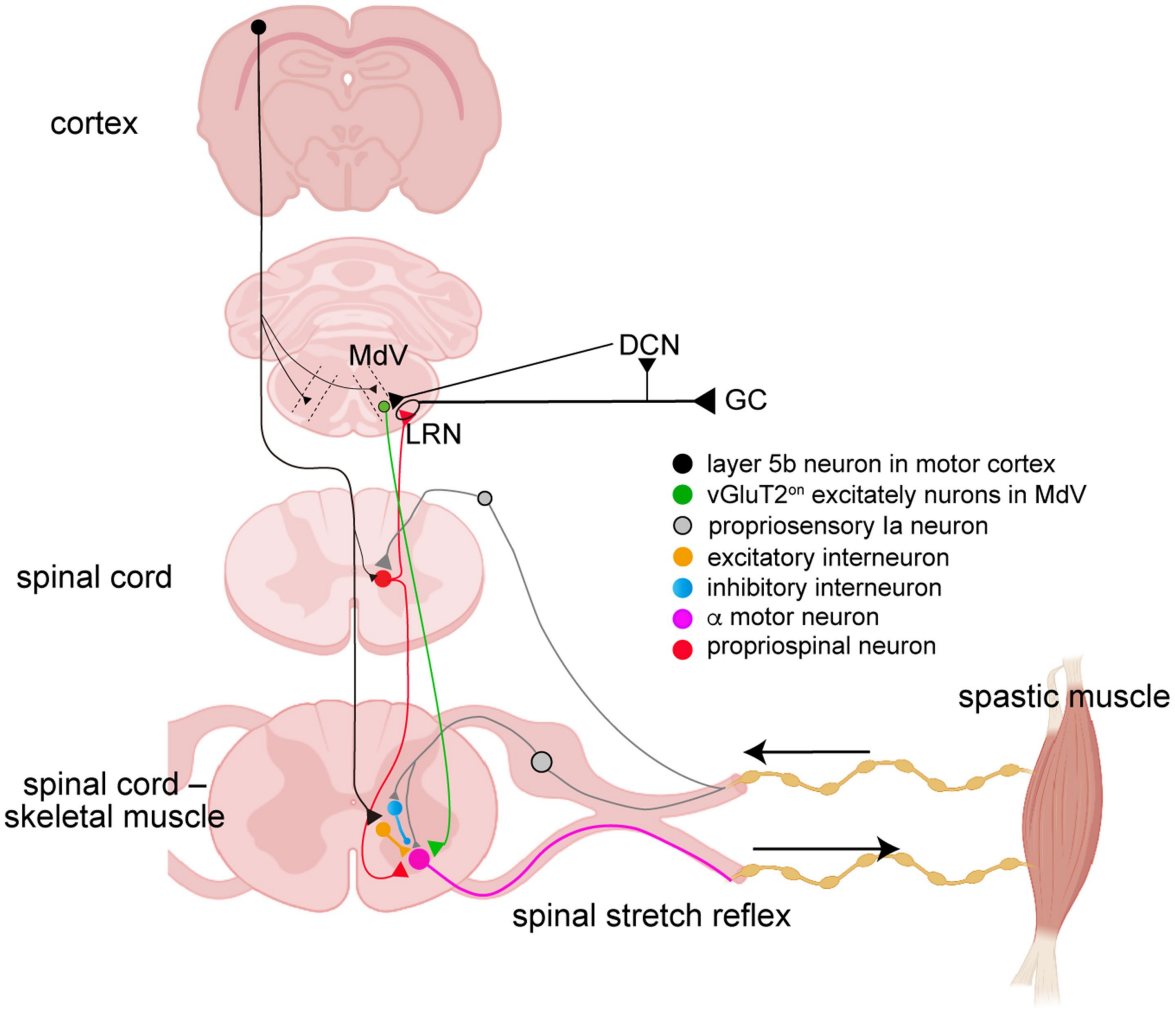
Overview of Ia afferent inputs to the medullary reticular formation via the lateral reticular nucleus and cerebellum. The Ia axon projects to the propriospinal neurons, which are bipolar neurons that connect the monosynaptic and motor neurons. The Ia axon-propriospinal neuron-LRN pathway is a candidate pathway for ascending sensory (Ia afferent) signals to the MdV. MdV, medullary reticular nucleus, ventral part; LRN, lateral reticular nucleus; GC, cerebellar granule cells; DNC, deep cerebellar nuclei.

**Figure 6:**
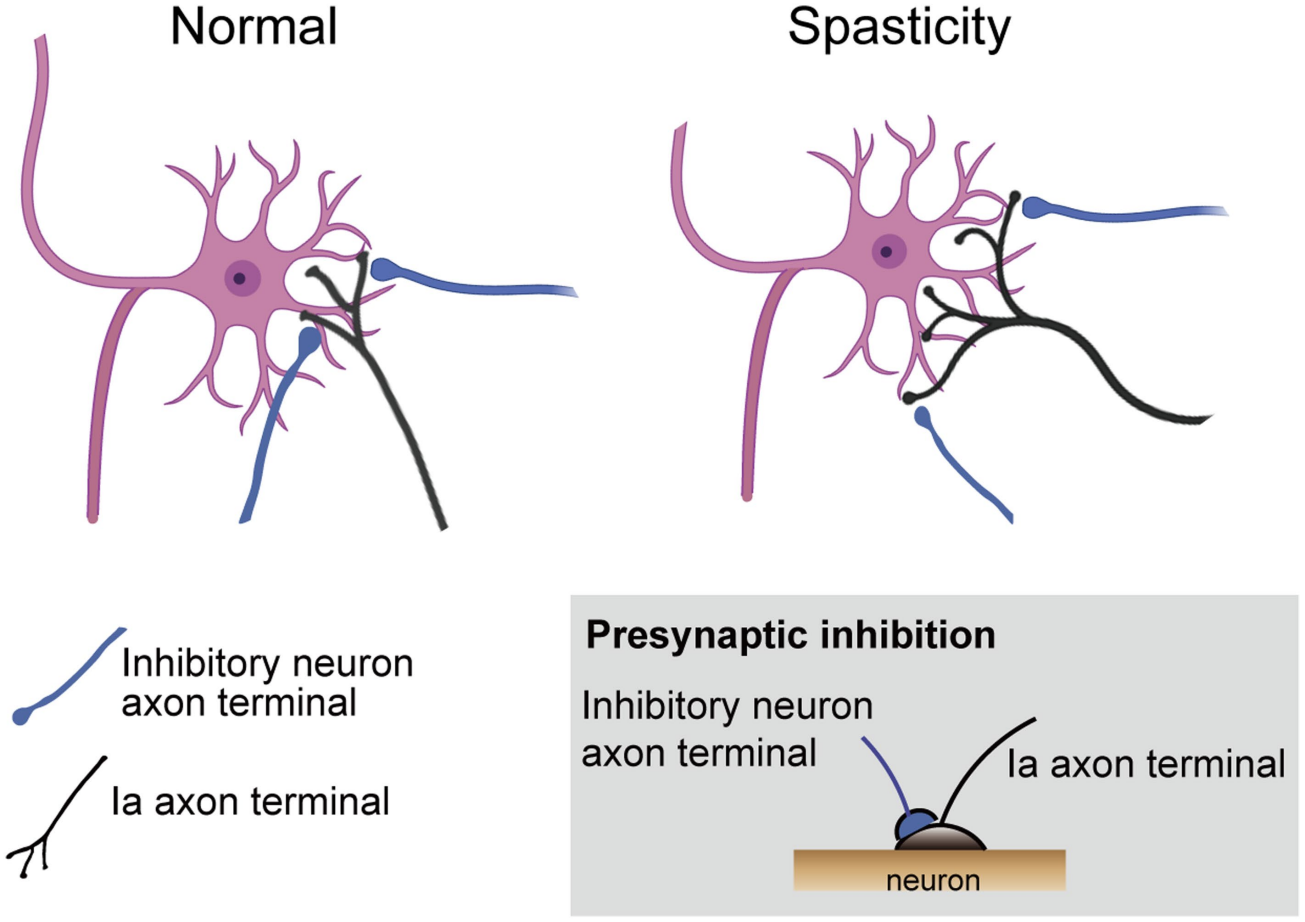
Overview of increased Ia input in spasticity, illustrated via comparative schematic of lateral axon branching in normal and spastic conditions. The schematic diagram of presynaptic Ia inhibition shows that inhibitory synapses connect at the axon terminal but not the neuronal cell body. Presynaptic inhibition controls the incoming information.

We recently reported that neuronal activity in the MdV following photothrombotic stroke was upregulated using quantitative activation-induced manganese-enhanced magnetic resonance imaging (Isumi et al., 2024). We hypothesized that the skillful movement of the hand of stroke individuals with spasticity is severely impaired because maladaptive plastic changes occur in the brainstem, especially in the MdV, which is vital for grasping actions (Esposito et al., 2014). The MdV neurons receive projections from the primary, premotor, and supplementary motor cortices ipsilaterally with a descending, monosynaptic connection to spinal motor neurons. They are almost entirely glutamatergic (excitatory) neurons (vGluT2^ON^, 93 ± 1%) that excite the forelimb premotor neurons (Esposito et al., 2014). Quantitative activation-induced manganese-enhanced MRI enables cumulative quantification of cellular activity using Mn^2+^ as a contrast agent with similar characteristics to calcium ions as a divalent cation. The Mn^2+^ concentration is quantified by measuring the absolute longitudinal relaxation time (T1, R1 = T1^−1^) of H^+^. That is, the higher the cellular activity, the closer the R1 value approaches one. We confirmed the changes in neural activity in the MdV using the R1 value of quantitative activation-induced manganese-enhanced MRI following stroke-onset spasticity in mice. The activity of contralesional MdV neurons, which project to the affected cervical motor neurons, increased significantly one-week post-stroke in mice. Furthermore, in experiments in which the Hoffman reflex was evoked via electric stimulation of afferent nerves to confirm the relevance of spasticity, activity in the contralesional MdV region was upregulated compared with the sham group up to the fourth week post-stroke. Interestingly, when sham mice were subjected to electrical stimulation, the activity of MdV cells in the contralesional region of stimulated sham mice was reduced compared with that in non-stimulated sham mice. Following a stroke, neuronal activity in the MdV exhibits abnormal responses that oppose the plastic changes during ascending transmission of sensory inputs from afferent nerves. The candidate pathway for ascending sensory signals reaching the MdV is expected to involve the propriospinal neurons and the lateral reticular nuclei (LRN) via the cerebellar nuclei (Figure 5). Ia axons connect to the propriospinal neurons, which are bipolar neurons that connect both motor neurons and the LRN. In the pathophysiology of spasticity, Ia axons sprout and the lateral branches increase (Figure 6) (Toda et al., 2014; Hanasaki et al., 2025). Therefore, the Ia axon endings and the number of Ia-a motor neuron connections also increase (Toda et al., 2014; Hanasaki et al., 2025). If the Ia axon endings connecting the propriospinal neurons increase, the ascending sensory inputs would be enhanced. Moreover, Ia axon terminals are regulated by presynaptic inhibition, which limits the amount of peripheral information entering the spinal cord. However, a reduction in presynaptic Ia inhibition has been reported to be associated with increased spasticity (Aymard et al., 2000; Achache et al., 2010). Taken together, these findings suggest that sensory afferent inputs to spinal and supraspinal circuits may be enhanced under spastic conditions. Thus, it is anticipated that inputs to the MdV in the reticular formation would decrease from cortical motor neurons and increase from afferent sensory neurons. Further clarification of the detailed mechanism underlying this shift is required. At the cellular level, such sustained alterations in synaptic input may engage intrinsic regulatory processes within MdV neurons. In addition, we clarified that one potential mechanism underlying the increased excitability of MdV neurons involves homeostatic plastic changes (Isumi et al., 2024). Homeostatic plasticity represents an intrinsic regulatory system that stabilizes neuronal activity by adjusting synaptic strength in response to sustained alterations in synaptic input. Specifically, neurons receiving altered afferent input undergo compensatory plastic changes to maintain stable levels of activity, often through changes in the expression of postsynaptic receptors. In this context, the present study reports alterations in the expression of a-amino-3-hydroxy-5-methyl-4-isoxazolepropionic acid (AMPA) receptor subtypes, which have been described as a hallmark of homeostatic plasticity.

## 8 Improvement of function or cause of spasticity?

Changes in neural plasticity within living organisms are generally regarded as a part of the recovery process following CNS injury. This review summarizes how such plastic changes contribute to the partial recovery of motor function and muscle strength that lost following stroke or spinal cord injury. Crucially, this recovery does not reflect the regeneration of the damaged neural structures themselves, but rather the reorganization and reconstruction of alternative neural circuits that compensate for the lost function. Consequently, although specific aspects of motor function may improve, certain movements remain difficult to recover. Particularly in primates, the recovery of skilled movement in the fingers appears to be limited when relying solely on compensatory circuit reorganization. Conversely, the reconstruction of alternative neural circuits can lead to the onset of spasticity. This phenomenon has been reported in both humans and non-human primates (Zaaimi et al., 2012; Xu et al., 2017). Thus, plastic changes within the brainstem reticular formation may represent a double-edged mechanism that contribute to partial motor recovery while simultaneously participating in the pathophysiology of spasticity. The brainstem reticular formation is not a homogeneous population of neurons but a complex assemblage comprising multiple nuclei with diverse functional characteristics. This review distinguishes between the medial and lateral pathways as defined in the clinical context, with a particular focus on the reticulospinal tract. Based on the available evidence in primates, especially humans and monkeys, neuroplastic changes in the medial pathway, primarily originating from pontine reticular formation, appear to be associated with enhanced facilitatory influences (Rhines and Magoun, 1946). In contrast, in rodents, the lateral tracts, which arise mainly from the medullary reticular formation, constitute the primary functional component of the reticulospinal system. Plastic changes predominantly involve these lateral tracts, and may exert both facilitatory and inhibitory effects.

Therefore, with regard to the spasticity and partial improvement in motor function arising from these plastic changes, we questioned whether spasticity reduction and functional improvement coexist? If spasticity develops because this response becomes excessive or inappropriate changes become fixed, such as inhibiting the formation of alternative neural circuits that would otherwise support functional improvement, early intervention to prevent such maladaptive outcomes may be the key to both reducing spasticity and enhancing function. Furthermore, rehabilitation and other interventions would likely be required to promote the construction of alternative corticospinal pathways that approximate the lost neural circuits.

Recent studies using rodent models have revealed that the brainstem reticular formation contains functionally specialized motor networks. Motor behaviors, such as speed regulation, directional control, and grasping movements, are generated through outputs from functionally specialized subcircuits within the brainstem. These findings challenge the traditional hierarchical model, in which motor control is primarily organized by descending commands from the cerebral cortex. Instead, accumulating evidence suggests that the reticular formation contains functionally organized networks that regulate specific motor modules.

A more detailed overview of this concept, provided in the review by Arber and Costa (2022). Importantly, these regulatory mechanisms are thought to involve not only the cerebral cortex and brainstem reticular formation, but also the basal ganglia and cerebellum, which together form parallel and bidirectional networks. From this perspective, functional recovery and the pathophysiology of spasticity after CNS injury should not be interpreted solely as abnormalities in the activity of specific nuclei within the brainstem reticular formation. Rather, they should be considered as consequences of the reorganization of multiple interconnected networks following disruption—including functionally specialized subcircuits and cortical networks.

## 9 Discussion

This review summarizes the plastic changes in the reticulospinal tract, with a specific focus on the pathophysiology of spasticity after stroke and SCI, integrating evidence from studies in humans, primates, and rodents. By systematically comparing the similarities and differences between stroke and SCI, as well as between species, plastic changes in the reticulospinal tract can be interpreted as a contributing mechanism to the onset of spasticity.

From a mechanistic perspective, an additional question is whether plasticity in the reticulospinal tract influences plasticity in other regions. Following stroke or SCI, alterations in neuronal activity and neural circuitry can also occur within the spinal cord, and such changes have been described in studies addressing the spinal-level pathophysiology of spasticity. However, it remains unclear whether these spinal plastic changes arise as a consequence of plasticity of the reticulospinal tract or are directly attributable to tissue damage caused by stroke or SCI. Our experimental findings suggest a possible interaction between these forms of plasticity. In our study, sensory stimulation elicited abnormal cellular responses in MdV of stroke-injured mice compared with uninjured mice (Isumi et al., 2024). This observation may be interpreted as indicating that plastic changes in sensory pathways, spinal circuits, and the reticulospinal system interact with and reciprocally influence each other. These findings raise the possibility that spasticity emerges from the reorganization of distributed motor networks rather than from plasticity within a single pathway. Furthermore, understanding how these network-level interactions contribute to the development of spasticity may provide important insight into the development of therapeutic strategies targeting neural circuit reorganization after CNS injury.
